# Study protocol for a randomised, patient- and observer-blinded evaluation of P6 acustimulation for the prevention of nausea and vomiting in the postoperative period in patients receiving routine pharmacological prophylaxis (P6NV-Trial)

**DOI:** 10.1186/s13063-022-06369-8

**Published:** 2022-06-16

**Authors:** Benedict Weber, Selena Knoth, Peter Kranke, Leopold Eberhart

**Affiliations:** 1 Department of Anaesthesiology and Intensive Care, Philipps-University of Marburg, Marburg, Germany; 2 Department of Anaesthesiology and Intensive Medicine, Asklepios Stadtklinik, Bad Wildungen, Germany; 3 Department of Anaesthesia and Critical Care, University Hospitals of Würzburg, Würzburg, Germany

**Keywords:** Postoperative nausea and vomiting, PONV, Antiemetics, Acupuncture, Acustimulation, Pericadium 6, P6, PC6, Multimodal therapy, Combination therapy

## Abstract

**Background:**

The efficacy of pericardium 6 (P6) acupoint stimulation to reduce the incidence of postoperative nausea and vomiting (PONV) has been proven in several randomised controlled clinical trials. However, little is known about the effectiveness in daily practice and its use in combination with traditional pharmacologic approaches.

**Methods:**

The primary objective of the P6NV study is to determine whether intraoperative acustimulation (acupuncture or acupressure) at the point P6 provides additional benefit when applied along with customary prophylactic intravenous antiemetics administered according to the local standard operating procedures (SOP). The primary endpoint is the incidence and severity of PONV within the first 24 h postoperatively reported with a validated postoperative nausea and vomiting intensity scale. The patient-reported outcome of perioperative quality of life (using the PPP33-questionnaire) and the detection of antiemetic-related side effects as well as the severity of PONV (via a standardised questionnaire) are secondary study objectives. P6NV is a national, multicentre, randomised, prospective, patient- and examiner-blinded interventional study and will be performed on 3500 adult patients with ASA classification I–III undergoing elective surgery under general anaesthesia and hospitalised for at least 24 h. Participating anaesthesiologists commit themselves to administer customised conventional antiemetic prophylaxis according to the local SOP by signing a statement before randomisation. The intervention group receives bilateral acupuncture or acupressure at P6. The control group receives no intervention. Before extubation, acustimulation is removed.

**Discussion:**

Since P6 acustimulation is performed by a wide range of anaesthesiologists receiving written and verbal information on acustimulation beforehand, this trial will provide information on the effectiveness of an ad hoc implementation of P6 stimulation techniques in anaesthesia departments using traditional pharmacologic PONV prophylaxis.

**Trial registration:**

DRKS DRKS00015272. Registered on August 15, 2018.

**Supplementary Information:**

The online version contains supplementary material available at 10.1186/s13063-022-06369-8.

## Administrative information

Note: The numbers in curly brackets in this protocol refer to SPIRIT Checklist item numbers. The order of the items has been modified to group similar items (see http://www.equator-network.org/reporting-guidelines/spirit-2013-statement-defining-standard-protocol-items-for-clinical-trials/).

**Table:** 

Title {1}	Study protocol for a randomised, patient- and observer-blinded evaluation of P6 acustimulation for the prevention of nausea and vomiting in the postoperative period in patients receiving routine pharmacological prophylaxis (P6NV-Trial)
Trial registration {2a and 2b}.	DRKS00015272
Protocol version {3}	Version 1.0 – 05.12.2017
Funding {4}	Only institutional funding was used to conduct this trial. Material for acustimulation was provided by Go Travel (Design Go Inc., 1800 NW Corporate Blvd., Suite 302, Boca Raton, FL 33431 USA) and 3B Scientific (3B Scientific GmbH, Ludwig-Erhard-Str. 20, 20459 Hamburg).
Author details {5a}	Benedict Weber: Philipps-University of Marburg, Department of Anaesthesiology and Intensive Care, Marburg, Germany Selena Knoth: Philipps-University of Marburg, Department of Anaesthesiology and Intensive Care, Marburg, Germany, Asklepios Stadtklinik, Department of Anaesthesiology and Intensive Medicine, Bad Wildungen, Germany Peter Kranke: Department of Anaesthesia and Critical Care, University Hospitals of Würzburg, Germany Leopold Eberhart: Philipps-University of Marburg, Department of Anaesthesiology and Intensive Care, Marburg, Germany
Name and contact information for the trial sponsor {5b}	Leopold Eberhart, Philipps-University of Marburg, Department of Anaesthesiology and Intensive Care, Baldingerstraße, 35033 Marburg
Role of sponsor {5c}	Investigator-initiated trial. Material for acustimulation was provided by Go Travel and 3B Scientific with no involvement in any stage of planning, conduction, or reporting of the trial.

## Introduction

### Background and rationale {6a}

Postoperative nausea and vomiting (PONV) is a frequent side effect of general anaesthesia. The incidence of PONV in untreated patients ranges from 10% (Apfel Score 0) up to about 80% (Apfel Score 4) [1, 2]. Often referred to as a big ‘little’ problem [3], severe PONV can result in major medical complications (e.g. wound dehiscence, aspiration of gastric contents, oesophageal rupture, pneumothorax, subcutaneous emphysema, and loss of vision [4, 5]). PONV is a leading cause of dissatisfaction with anaesthesia [6–8]. Not surprisingly, patients are willing to pay up to US $100 for a completely effective therapy [9–11]. Furthermore, it can be an economical issue due to a prolonged stay in the recovery room after surgery, further antiemetic medication, extra nursing time, delays in discharge, and, finally, unplanned readmission in the outpatient setting [11–14].

Administering antiemetic drugs avoiding volatile anaesthetics [15] and nitrous-oxide effectively reduce the incidence of PONV. These effects are additive [16]. A Cochrane review found evidence that pre- and intraoperative stimulation at pericardium 6 (P6) compared to a dummy procedure is also an effective intervention for preventing PONV [17]. There are also a few studies comparing acupuncture at P6 to a single antiemetic drug (e.g. dexamethasone [18], prochlorperazine [19]), showing positive results of P6.

The consensus guidelines for the management of PONV also suggest acupoint stimulation for the prophylaxis of PONV. However, little is known about the effectiveness in daily practice and the usefulness of this therapeutic option being used along with conventional pharmacologic approaches to prevent postoperative nausea and vomiting [20].

### Objectives {7}

We designed this pragmatic clinical trial to provide evidence whether ad hoc implementation of techniques for P6 acustimulation is effective to improve conventional pharmacologic prophylaxis administered on a customary basis in accordance with local standard operating procedures (SOP) in adult inpatients undergoing elective surgery.

### Trial design {8}

The study is designed as a multicentre, randomised, prospective, patient- and observer-blinded interventional trial.

## Methods: participants, interventions, and outcomes

### Study setting {9}

The study is conducted after positive follow-up votes from the ethics committees of the participating hospitals following a positive vote from the leading ethics committee of the Philipps-University of Marburg (chairman: Prof. Dr. G. Richter; approved on March 6, 2018 (Az.209/17)). The trial was registered at DRKS with the identifier: DRKS00015272. The study protocol will be performed in accordance with the Declaration of Helsinki and the ICH-GCP guidelines.

Participating clinics are intended to be representative of the German hospital landscape. Currently, five hospitals recruit patients to this study (one university hospital, one clinic of maximum treatment, three clinics of standard care, full list available from the authors). One main goal was to reduce interference with routine care for patients in participating centres as much as possible. A full list of study sites can be obtained from the sponsor.

### Eligibility criteria {10}

#### Inclusion criteria

The following are the inclusion criteria:

- 18 years of age or older
- Ability to participate in the postoperative assessment for PONV
- Scheduled for general anaesthesia
- Inpatients expected to be hospitalised for at least 24 h after surgery

#### Exclusion criteria

The following are the exclusion criteria:

- Pregnancy and/or lactation
- Expected need for postoperative ventilation and/or intensive care
- Antineoplastic therapy, chemotherapy, or radiotherapy within the past 4 weeks for patients experiencing preoperative nausea

Reasons for dropout will include retraction of patient’s consent before or during participation, unexpected transfer to intensive care unit with intubation, ventilation, and sedation during follow-up.

### Who will take informed consent? {26a}

Patients are addressed in the preoperative assessment clinic and are provided with information on the aim of the study by the anaesthesia staff of participating clinics.

### Additional consent provisions for collection and use of participant data and biological specimens {26b}

Informed written consent is obtained from all participating patients meeting all inclusion and none of the exclusion criteria.

### Interventions

#### Explanation for the choice of comparators {6b}

Because of the nature of the intervention (acustimulation), no intervention will be performed in the control group.

#### Intervention description {11a}

##### Preparation for anaesthesia

The study protocol does not interfere with the preoperative preparation of patients. Consequently, patients may receive routine oral premedication the evening before and the morning of the surgical procedure. After entering the operating theatre, the usual procedures are performed (e.g. WHO Safety Checklist, standard monitoring, iv catheter). Induction and maintenance of anaesthesia are performed according to routine practice. All drugs administered due to the surgical procedure (e.g. premedication, induction agents, opioids, muscle relaxants) are recorded with their dosages and route of application, respectively.

##### Pharmacological antiemetic prophylaxis

The study aims to identify the effectiveness of P6 acustimulation along with conventional antiemetics administered intraoperatively. Thus, there is a high risk of bias that techniques for P6 stimulation might interfere with the pharmacological measures against PONV and vice versa. Special efforts are undertaken to ensure unbiased and comparable use of antiemetics in both groups. Comprehensive information is provided to the anaesthesiologists which highlight the need for ‘usual care’ in both groups. Additionally, all anaesthesiologists are required to fill in a document for personal ‘self-commitment’. With this form, the anaesthesiologist determines and documents the antiemetic prophylaxis that is suitable and will be administered to the individual patient (for the form, see Additional file 1). This part of the documentation form is filled out and signed by the anaesthesiologist and the attending nurse (four-eyes principle) before group allocation is revealed. This allows the determination of pharmacological interventions against PONV independent of the result of the randomisation procedure.

##### Antiemetic medication

The anaesthesia staff administers antiemetics according to their self-committed individual medication plan. One of a combination of the following measures may be used:

- ▪ Dexamethasone 4–8 mg
- ▪ 5-HT3-antagonists (e.g. ondansetron 4–8 mg, granisetron 1 mg)
- ▪ Droperidol 0.5–1.5 mg
- ▪ Haloperidol 0.5–1.5 mg
- ▪ Metoclopramide 10–20 mg
- ▪ Dimenhydrinate 50–70 mg
- ▪ Fosaprepitant (no dose restriction)
- ▪ Use of propofol for total intravenous anaesthesia
- ▪Omission of nitrous oxide

The attending anaesthesiologist is free to administer any of the above-mentioned interventions and to combine drugs of different pharmacologic classes (e.g. due to high individual risk of the patient for PONV) but also to withhold antiemetic medication (e.g. if a low risk for PONV is present). Furthermore, the time of administration can be chosen by the overseeing anaesthesia team. The chosen prophylaxis (drug(s), dose, and time of administration) is recorded in a paper-based case report form (CRF).

##### Intervention

The anaesthesia team is allowed to open the opaque randomisation envelope after completing the self-commitment. This envelope includes information whether the patient is allocated to the control or the intervention group.

Patients allocated to the intervention group receive acustimulation at both forearms at the P6 point immediately after the induction of anaesthesia. Pressure is applied to the needles for about 15 s at the beginning and the end of the intervention to stimulate P6. The applied force should cause the needle tip to reach a depth of 0.5 to 1 cun (traditional Chinese measuring unit) [21]. Anaesthesiologists can also use acupressure bands for intervention instead of acupuncture needles. The intervention and its duration of administration are recorded.

The control group receives only the designated antiemetic prophylaxis according to the self-commitment.

**Fig. 1:**
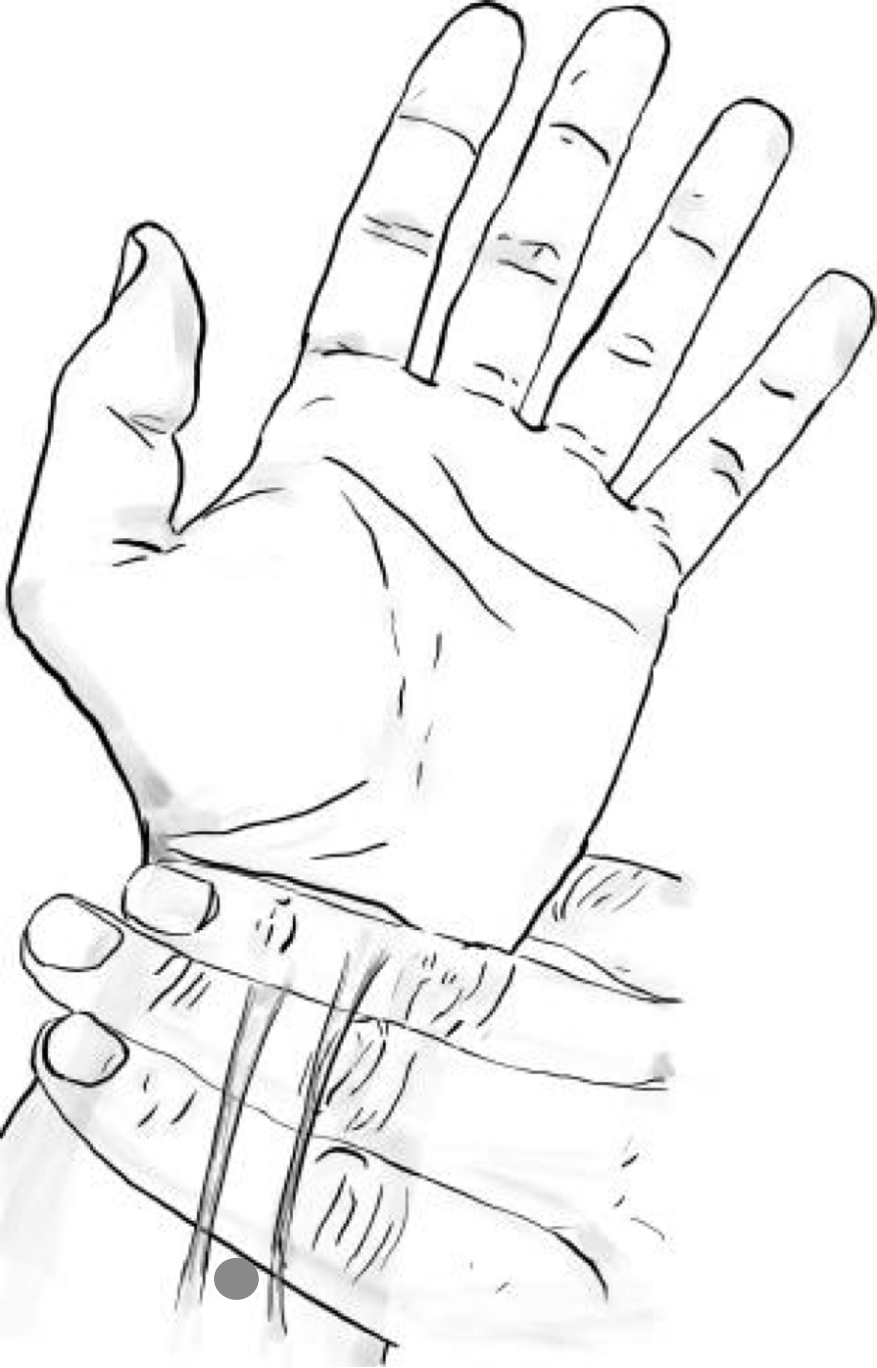
Location of pericardium 6 (P6). The illustration is used to train the staff. The grey dot indicates the position of P6 between the flexor carpi radialis and the palmaris longus tendons

The P6 acupoint is located 2 cun [22] from the distal palmar crease between the flexor carpi radialis tendon and the palmaris longus tendon on the volar surface of both forearms [23]. All anaesthesiologists were informed about the study and were provided with verbal and written information on anatomical location of the P6 stimulation point and the need for applying pressure to the needle. An information sheet (including the illustration from Fig. 1) was available in all operating rooms. For acupuncture, Seirin® New Pyonex needles with a length of 1.5 mm will be used. Acupressure is performed with GoTravel Acustrap bands.

P6 stimulation ends right before emergence from anaesthesia. Opaque dressings are applied at the (potential) sites of stimulation in both groups to ensure patient and observer blinding.

##### Postanaesthesia care unit (PACU)

Postoperatively, patients are transferred into the PACU. Treatment of postoperative pain and other symptoms is performed according to usual care but documented in the CRF. Nausea and vomiting and potential antiemetic medication administered in the PACU are of special interest.

##### Ward (first postoperative day)

On the first postoperative day (22–26 h after the end of anaesthesia), patients are visited by a member of the study team. This person is blinded to the type of treatment and without knowledge of the anaesthesia technique performed or perioperative medication administered. Patients receive questionnaires targeting the occurrence of nausea and vomiting and overall well-being. Patients are asked to complete these forms by themselves, but help can be provided if necessary.

#### Criteria for discontinuing or modifying allocated interventions {11b}

The intervention will take place during anaesthesia. If PONV is occurring afterwards, routine care will be performed and documented.

#### Strategies to improve adherence to interventions {11c}

Antiemetic prophylaxis and therapy are documented as described before. The anaesthesiologists’ self-commitment is used to minimise bias regarding the antiemetic therapy. Any deviations from the specified prophylaxis are documented, stating the reason for the deviation. Anaesthesiologists are trained with a standardised procedure (see Fig. 1) to avoid poor implementation of P6 stimulation and insufficient stimulation time of P6.

#### Relevant concomitant care permitted or prohibited during the trial {11d}

n/a: Routine care is performed.

#### Provisions for post-trial care {30}

To the best of our knowledge, there is no harm documented in the literature by the materials used for acustimulation.

### Outcomes {12}

#### Primary endpoint

The primary outcome measure for this study is the incidence of PONV within a 24-h observation period. PONV is defined as nausea, retching, vomiting, or the need for any antiemetic medication during the observation period.

#### Secondary endpoints

Secondary endpoints include the following

1. The intensity of PONV during the 24 h observational period is classified using the ‘postoperative nausea and vomiting Intensity Scale’ (PIS) [24],
2. The patient-reported outcome of perioperative quality of life.
3. The incidence and severity of adverse reactions caused by the different combinations of antiemetic drugs.

These endpoints are determined by using the following specific questionnaires:

- ▪ ‘Patient Evaluation in the Perioperative Phase’ (PPP33)
- ▪ Adverse effects via the ‘Postoperative side effects questionnaire’ (PON-F)

### Participant timeline {13}

The participant timeline is presented in the following table.

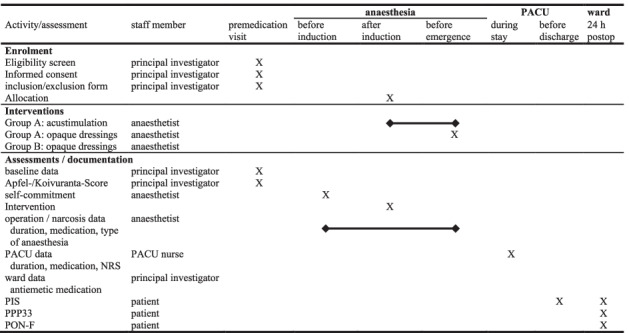



### Sample size {14}

Sample size calculation assumes a baseline incidence of PONV of 25%. This number is lower than reported in surveys on PONV with 29% [25] but considers routine pharmacological prophylaxis, at least in patients with increased risk for PONV.

A Cochrane review by Anna Lee [17] suggests that P6 stimulation can reduce PONV by approximately 30% (relative risk reduction), leading to a PONV-incidence in the treatment group of 17.5%. The trial is powered to detect an absolute reduction of 5% points (from 25 to 20%). This conservative estimation of P6 treatment considers that efficacy will not be as perfect as in efficacy trials, e.g. due to inconsequent or erroneous administration of the P6 stimulation. Power analysis was performed with PASS 2002 (Number cruncher software) and revealed that 1503 patients provide a power of 80% to detect an absolute 5% point difference with a type I error of 5% using the two-sided Fisher’s exact test. With an added safety margin of about 15% compensating for dropouts, etc. 2 × 1750 patients must be included.

### Recruitment {15}

Recruitment will be performed by anaesthesia staff of the participating clinics during usual preoperative visits.

### Assignment of interventions: allocation

#### Sequence generation {16a}

Randomisation is performed as block randomisation with permuted blocks of variable length by staff members of the coordinating study centre. Random numbers are obtained from www.random.org. Each individual set of randomisation envelopes contains 50% of each group for equal distribution of the groups per centre. An Excel® Spreadsheet was created with 45 sets of 100 randomisations each. For each set, a random number of blocks with a random and even-numbered length were created.

#### Concealment mechanism {16b}

Opaque envelopes are sent to the participating clinics. These contain the case report form and the group allocation. Measures are adopted to prevent access from unauthorised persons.

#### Implementation {16c}

The allocation sequence with random IDs in ascending order is generated by staff members of the coordinating study centre and assigned to the opaque randomisation envelopes. Patients are enrolled by the study staff in the participating clinics during the preoperative visit and assigned by using the randomisation envelopes in ascending order.

### Assignment of interventions: blinding

#### Who will be blinded {17a}

This study is designed patient- and observer-blinded. The intervention is applied only during the maintenance of general anaesthesia. Thus, the patient remains blinded to the intervention. All patients receive opaque dressings on the intervention sites on both forearms. The patient’s group assignment and intervention remain hidden from the PACU nurses as they gather the primary endpoint as well as the staff that collects data during the follow-up. Data analysis will be performed blinded as well.

#### Procedure for unblinding if needed {17b}

Unblinding of the intervention is permitted after contacting the coordinating study centre if (serious) adverse events occur.

### Data collection and management

#### Plans for assessment and collection of outcomes {18a}

Baseline data (gender, age, risk factors for PONV, etc.) is recorded during the preoperative visit. Additional information is recorded during the anaesthetic procedure including the administered medication. Outcomes and medication are assessed during the stay in the postanaesthesia care unit and on the first postoperative day in the ward by the anaesthesia staff of the participating clinics. These include the following tools.

The Postoperative Nausea and Vomiting Intensity Scale (PIS) provides a clinical evaluation of nausea, retching, and vomiting and allows for differentiation between transient and mild nausea symptoms and clinically relevant PONV (see Additional file 2) [24]. The nursing staff fills in the PIS in the PACU and patients themselves complete the PIS on the first postoperative day.

PPP33 is a questionnaire measuring the perioperative quality of life (see Additional file 3). It contains 33 questions. Answers to individual questions are valued from 1 to 4. An overall score—containing eight sub-scales representing eight different dimensions (information, fear, autonomy, pain, physical complaints, rest, communication, accommodation) of the perioperative quality of life—can be calculated. Higher scores indicate a higher quality of life [26, 27].

PON-F is used for the assessment of side effects of antiemetic drug combinations (see Additional file 4). PON-F consists of 38 items representing symptom descriptions organised by body regions. The symptoms and the physical constraints caused by these symptoms can be rated as ‘severe’, ‘moderate’, ‘mild’, and ‘not present’. Additionally, the patient must decide whether the symptom is related to anaesthesia. The objective is to ascertain the antiemetic-related side effects with a focus on their incidence and the simultaneous administration of various antiemetics.

#### Plans to promote participant retention and complete follow-up {18b}

The study staff assesses the outcomes until the first postoperative day. Administered antiemetic medication is documented as it can be obtained from the patient record.

#### Data management {19}

Data collection is facilitated using a paper-based case report form (CRF) and fed into a web-based database by the investigators. Additionally, the data collection includes baseline data gathered during the admission interview, the anaesthesiologist’s intended antiemetic prophylaxis, and data on anaesthesia management, the course in the recovery room, and the need for antiemetic rescue medication in the first 24 h. The CRF is supplemented by patient-related outcomes form consisting of a three-page questionnaire containing PIS, PPP33, and PON-F filled out by the patients on the first postoperative day.

##### Database infrastructure

The web-based database was created using typical web standards and is hosted by the university computer centre of Philipps-University of Marburg. Access is restricted to study staff by login. Input formats are validated, and an audit trail was established to ensure data integrity. Coding of data is performed after the submission of the data form. The corresponding coding can be found on the paper-based CRF. Regular backups of the database are performed.

#### Confidentiality {27}

Pseudonymisation is ensured by using the corresponding ID in the database. The ID is linked to the clinic’s patient ID by lists which are maintained in the study centres.

#### Plans for collection, laboratory evaluation, and storage of biological specimens for genetic or molecular analysis in this trial/future use {33}

n/a: No biomaterial obtained

## Statistical methods

### Statistical methods for primary and secondary outcomes {20a}

Statistical evaluation is performed using RStudio version 1.2 (RStudio Team (2018): Integrated Development for R. RStudio, Inc., Boston, MA. URL http://www.rstudio.com/). Statistical significance is generally assumed at a *p*-value < 0.05.

To check for homogeneity between the two groups, the Pearson *χ*^2^-test is used for nominal scale levels. Student’s *t*-test for independent samples is used for continuous data after performing the Shapiro-Wilk test and the Levene test. The non-parametric equivalent is the Mann-Whitney *U* test.

Metric data are reported as the arithmetic mean, standard deviation, or median with 25% and 75% quantile. Ordinal data is given as median with 25% and 75% quantiles.

Scores of the tools used for assessing the outcome are calculated as intended by tools’ authors.

### Interim analyses {21b}

We plan to perform an interims analysis after recruitment of 50% of patients (*n* = 1750 patients). It is intended to use the secondary endpoint ‘severity of PONV’ since this is a suitable indicator for the primary endpoint ‘incidence of PONV’. A threshold of a *p*-value ≥ 0.1 is defined as an indicator of futility and will cause the termination of the study. If the *p*-value is below this threshold, the study will be completed as planned.

### Methods for additional analyses (e.g. subgroup analyses) {20b}

Logistic regression will be performed for subgroup analysis concerning group assignment, preoperative risk of PONV and administered antiemetic medication and their effects on PONV.

### Methods in analysis to handle protocol non-adherence and any statistical methods to handle missing data {20c}

Calculations will be performed as intention-to-treat analysis and per-protocol analysis. Imputation with the EM algorithm using the R-package ‘Amelia’ will be performed after missing data analysis.

### Plans to give access to the full protocol, participant-level data, and statistical code {31c}

Additional information can be obtained from the principal investigator.

### Oversight and monitoring

#### Composition of the coordinating centre and trial steering committee {5d}

The coordinating study centre holds the staff for support of the participating clinics. During recruitment, quarterly meetings are set to ensure constant recruitment and provide solutions for occurring problems.

#### Composition of the data monitoring committee, its role, and reporting structure {21a}

n/a: Investigator-initiated trial, no clinical trial under the German Medicines Act (‘AMG’).

#### Adverse event reporting and harms {22}

Adverse events are reported directly to the principal investigator by the staff of the participating clinic via a corresponding form.

#### Frequency and plans for auditing trial conduct {23}

Quarterly meetings will be held by the study staff of the coordinating centre including overseeing recruitment and submitted data of the participating clinics.

#### Plans for communicating important protocol amendments to relevant parties (e.g. trial participants, ethical committees) {25}

Any relevant changes to the protocol will be communicated directly by the principal investigator with any relevant party.

#### Dissemination plans {31a}

Publication is planned either after full recruitment or prior termination as stated in the criteria for interim analysis.

## Discussion

It is generally accepted that PONV can be reduced to a clinically satisfying level by applying a multimodal antiemetic approach. This can be achieved by combining drugs from different pharmacologic classes or adding the latter to a total intravenous anaesthesia technique. So far, there is limited evidence whether P6 stimulation can be integrated into such a multimodal antiemetic concept or if the addition of acustimulation results in a further reduced incidence of PONV [28–36].

This paper describes the methodology and clinical endpoints of a large randomised controlled clinical trial that investigated the use of acustimulation at point P6 in combination with an individually tailored pharmacological antiemetic treatment regarding the incidence of PONV. Emphasis is given to potential biases deriving from the lack of blinding and inhomogeneous administration of antiemetics to the treatment group versus the control group.

Since P6 acustimulation is performed by anaesthesiologists with limited training performing the procedure, this trial will provide information on the use of an ad hoc implementation of these techniques in an anaesthesia department under real clinical practice conditions. Therefore, the anaesthetic administration has not been standardised, and the results will be transferable to the largest possible patient population.

### Limitations of the study

Our study may be best classified as a patient- and observer-blinded, block-randomised pragmatic clinical trial. Many efforts were focused on documenting the intended prophylaxis of PONV before the allocation to the study group. For blinding purposes, acustimulation is only applied in the intraoperative period. This rather short-term stimulation during anaesthesia may limit the efficacy of the intervention. Additional emetogenic stimulus (e.g. administration of postoperative opioids) occur after termination of the acustimulation further limiting the effect of the P6 stimulation.

The baseline PONV management is not standardised which might cause an imbalance of baseline PONV risk. We address this particular risk by using risk calculation in both trial arms.

P6 stimulation for this study is based on common descriptions and opinions about traditional acustimulation. However, the study’s standardised procedure of P6 stimulation may be considered as insufficient in duration or technique overall. Therefore, the result can only describe this study’s approach of an ad-hoc implementation with a common standard of P6 stimulation rather than the general effectiveness of P6 stimulation.

### Trial status

Protocol version 1.0 (date 05.12.2017)

First patient in 01.09.2018

Approximate last patient in 31.12.2022

## Supplementary Information

**Additional file 1.** Self-commitment for intended prophylaxis. Self-commitment regarding the antiemetic prophylaxis and therapy as included in the case report form (CRF).

**Additional file 2.** Postoperative nausea and vomiting intensity scale (PIS). Own illustration as included in the case report form (CRF) based on Wengritzki et al. 2010 [24].

**Additional file 3.** Patient evaluation in the Perioperative Phase PPP33. Own illustration as included in the case report form (CRF) based on Eberhart et al. 2006 [26].

**Additional file 4.** Postoperative side effects questionnaire (PON-F). Own illustration as included in the case report form (CRF).

## Data Availability

The data is available to the study group. A full dataset can be obtained from the principal investigator by request.

## Abbreviations

ASA: American Society of Anesthesiologists
CRF: Case report form
P6: Pericardium 6
PACU: Postanaesthesia care unit
PIS: Postoperative Nausea and Vomiting Intensity Scale
PON-F: Postoperative Side Effects Questionnaire
PONV: Postoperative nausea and vomiting
PPP33: Patient evaluation in the Perioperative Phase
SOP: Standard operating procedures
