# Supplementary material for: Study protocol for a randomised, patient- and observer-blinded evaluation of P6 acustimulation for the prevention of nausea and vomiting in the postoperative period in patients receiving routine pharmacological prophylaxis (P6NV-Trial)
Source: Trials. 2022 Jun 16;23:497. doi: 10.1186/s13063-022-06369-8 (PMC9205120; doi:10.1186/s13063-022-06369-8)
Supplement: Supplementary file 2 — Additional file 2. Postoperative nausea and vomiting intensity scale (PIS). Own illustration as included in the case report form (CRF) based on Wengritzki et al. 2010 [24]. [file 13063_2022_6369_MOESM2_ESM.docx]

**Study protocol for a randomised, patient- and observer-blinded evaluation of P6 acustimulation for the prevention of nausea and vomiting in the postoperative period in patients receiving routine pharmacological prophylaxis (P6NV-trial).**

Benedict Weber^1^, Selena Knoth^1,2^, Peter Kranke^3^, Leopold Eberhart^1^

^1^Philipps-University of Marburg, Department of Anaesthesiology and Intensive Care, Marburg, Germany

^2^Asklepios Stadtklinik Bad Wildungen, Clinic for Anaesthesiology and Intensive Care, Bad Wildungen, Germany

^3^Department of Anaesthesia and Critical Care, University Hospitals of Würzburg, Germany

*Corresponding author*: Prof. Dr. Leopold Eberhart, Philipps-University of Marburg, Department of Anaesthesiology and Intensive Care, Baldingerstraße, 35033 Marburg, eberhart@staff.uni-marburg.de, +49 (0) 6421 586 6989

**Additional File 2: Postoperative nausea and vomiting intensity scale (PIS)**

Own illustration as included in the case report form (CRF) based on Wengritzki et al. 2010.

*Reference:*

Wengritzky R, Mettho T, Myles PS, Burke J, Kakos A. Development and validation of a postoperative nausea and vomiting intensity scale. Br J Anaesth. 2010;104:158–66.
