# Supplementary material for: Study protocol for a randomised, patient- and observer-blinded evaluation of P6 acustimulation for the prevention of nausea and vomiting in the postoperative period in patients receiving routine pharmacological prophylaxis (P6NV-Trial)
Source: Trials. 2022 Jun 16;23:497. doi: 10.1186/s13063-022-06369-8 (PMC9205120; doi:10.1186/s13063-022-06369-8)
Supplement: Supplementary file 4 — Additional file 4. Postoperative side effects questionnaire (PON-F). Own illustration as included in the case report form (CRF). [file 13063_2022_6369_MOESM4_ESM.docx]

**Study protocol for a randomised, patient- and observer-blinded evaluation of P6 acustimulation for the prevention of nausea and vomiting in the postoperative period in patients receiving routine pharmacological prophylaxis (P6NV-trial).**

Benedict Weber^1^, Selena Knoth^1,2^, Peter Kranke^3^, Leopold Eberhart^1^

^1^Philipps-University of Marburg, Department of Anaesthesiology and Intensive Care, Marburg, Germany

^2^Asklepios Stadtklinik Bad Wildungen, Clinic for Anaesthesiology and Intensive Care, Bad Wildungen, Germany

^3^Department of Anaesthesia and Critical Care, University Hospitals of Würzburg, Germany

*Corresponding author*: Prof. Dr. Leopold Eberhart, Philipps-University of Marburg, Department of Anaesthesiology and Intensive Care, Baldingerstraße, 35033 Marburg, eberhart@staff.uni-marburg.de, +49 (0) 6421 586 6989

# Additional File 4: Postoperative side effects questionnaire (PON-F).

Own illustration as included in the case report form (CRF).
